# Immigration, screening, and cervical cancer incidence: an application of Age–Period–Cohort analysis

**DOI:** 10.1097/CEJ.0000000000000495

**Published:** 2018-12-07

**Authors:** Dania Bucchi, Manuela Chiavarini, Fortunato Bianconi, Maria E. Galeotti, Alessio Gili, Fabrizio Stracci

**Affiliations:** a School of Specialization in Hygiene and Preventive Medicine; b Department of Experimental Medicine, Public Health Section, University of Perugia, Perugia; c Umbria Cancer Registry, Umbria, Italy

**Keywords:** Age–Period–Cohort, carcinoma *in situ*, cervical cancer, cervical dysplasia, human papilloma virus, immigration, inequalities, screening

## Abstract

Cervical cancer (CC) control is based on the implementation of effective screening programs. In the coming years, human papilloma virus vaccination coverage will contribute considerably toward cancer prevention. In Italy, where an organized screening program has been implemented, immigration from low/middle-income countries with a high prevalence of human papilloma virus infections has increased steadily over the last decades. To assess the impact of screening efforts in counteracting background changes, we analyzed the incidence trends of cervical intraepithelial neoplasia grade 3 carcinomas *in situ* (CIS) and invasive CC from 1994 to 2013 through an Age–Period–Cohort model using data of a regional population-based registry. Moreover, using Joinpoint regression, we compared the incidence of cervical lesions in native women with that observed in foreign-born women, highlighting the differences in age and screening status. The results indicate that the CC incidence trend decreased in Italian women (annual percent change = −2.7*%, 95% confidence interval = −4.3; −1.1), but increased (APC = 12.2*%, 95% confidence interval = 7.6; 17.0) in immigrants. For CIS, incidence rates show a growing trend in both groups, especially in women born abroad. For cancer, no marked changes in period-specific incidence rate ratios were detected until around 2000, when we found a slight decrease, followed by an increase. For CIS, we estimate an important upward trend in cohort-specific risks. The favorable effect of screening in preventing an increase in CC incidence has been counteracted by the progressive increase in immigrants from high-risk countries, where it is of increasing relevance to extend the use of vaccination.

## Introduction

Cervical cancer (CC) is the fourth most common female tumor worldwide and the fourth most common cause of cancer mortality among women.

There are considerable geographical differences in the global distribution of this disease: the large majority (about 85%) of the global burden occurs in low/middle-income countries, such as South America, Southeast Asia, Western, and Sub-Saharan Africa (Torre *et al.*, 2015). However, the incidence in many developed countries is low and the neoplasia is well controlled.

The incidence rates of CC correlate with the prevalence of human papilloma virus (HPV) infection in the population (Sharma *et al.*, 2013) and with the implementation of and participation in screening programs (Vaccarella *et al.*, 2016). In one study, the highest HPV prevalence was observed in Sub-Saharan Africa, Eastern Europe, and South America (Bruni *et al.*, 2010; Bray *et al.*, 2013), paralleled by the highest incidence and mortality for CC.

In Italy, where a free public screening program has been in place since the second half of the 1990s, mortality for CC has been decreasing steadily in the last two decades. CC represents about 1% of tumors in the female population older than 50 years of age and it is the fifth most common tumor in women younger than 50 years of age (AIOM, AIRTUM.I numeri del cancro in Italia, 2016).

Immigration is a relatively recent phenomenon in Italy that has been increasing constantly over the last decades and has led to significant changes in the composition of society. The overall percentage of migrants among the Italian population increased from less than 1% in 1991 to 7.4% in 2013 and continues to increase [Gli stranieri in Italia: analisi dei dati censuari, 2006; Resident foreigners (database online), 2014]. Most immigrants come from low-income/middle-income countries in Central and Eastern Europe, Asia, and North Africa, where there is a high HPV prevalence and a limited or no implementation of screening programs.

To assess the impact of screening efforts set against background changes, we analyzed the incidence trends both of CC (code C53, International Classification of Diseases - 10th Revision) and cervical intraepithelial neoplasia grade 3 (CIN III) carcinoma *in situ* (CIS) (code D06), investigating the underlying factors that affect incidence by an Age–Period–Cohort analysis. Furthermore, we examined the incidence of cervical lesions in Italian-born women compared with foreign-born women, highlighting the differences in age and screening status.

## Participants and methods

### Setting

Umbria is an Italian region with about 886 000 inhabitants as of January 2013; 28% of whom (about 250 000) were women in the target group for screening. Immigrant women accounted for about 11% of the female population; in particular, about 8% were between 25 and 64 years of age [Resident population (database online), 2014].

The screening program started in 1999, with all resident women aged 25–64 years invited for a Pap test every 3 years. Since 2014, a DNA-HPV testing has been offered every 5 years to women aged 35−64 years. Approximately 40% of the eligible population participated in organized screening (Osservatorio Nazionale Screening. Rapporto 2017), whereas about 30−40% underwent an opportunistic test (Rapporto nazionale Passi 2013: screening cervicale).

### Data source

Incidence data of CC were extracted from the Umbrian Population Cancer Registry (RTUP), a regional database established on 1 January 1994, that routinely collects information about the first diagnosis of cancer occurring in the resident population.

The organized screening program is monitored through a regional information system that registers and integrates data on invitations, cervical cytology, and histology.

### Study population

This study has taken into account all cases of CC and cervical CIN III/CIS diagnosed in Umbria between 1 January 1994 and 31 December 2013. Screening data, relative to Pap testing performed on women aged 25−64 years, refer to the period from 1 January 2001 to 31 December 2013.

### Study variables

Data was analyzed by age groups (25−64 years and ≥ 65 years) and by place of birth (Italian-born and foreign-born women) and in terms of screening status. Screen-detected CC included both diagnosed cases following participation in the regional screening program and cases identified with an opportunistic Pap smear; CIN III were categorized in cases diagnosed with opportunistic screening and in cases detected with organized screening.

### Statistical analysis

To make them comparable, the rates were standardizedw by age using the European standard population as of 2011 as a reference.

Descriptive statistics were calculated using frequencies, percentages, and frequency tables for categorical variables and medians and ranges for quantitative variables. Tests on the equality of proportions were performed using large-sample statistics (Wang, 2000).

The Joinpoint regression analysis was carried out to detect significant changes in cancer age-standardized incidence rates (Kim *et al.*, 2000) using the modified Bayes information criterion as the selection method (Zhang and Siegmund, 2007). The trends were characterized in terms of annual percent changes (APCs), with 95% confidence intervals (CIs) for each period. A *P* value less than 0.05 was considered statistically significant for all analyses presented.

To separate trends in terms of age, diagnosis period, and birth cohort effects, we used an extension of an Age-Period-Cohort model (Clayton and Schifflers, 1987), incorporating restricted cubic splines, implemented in Stata by Rutherford *et al.* (2010). The period effects are proxies for changes in CC risk in time (e.g. implementation of screening program), whereas the birth cohort effects reflect changes in sexual behavior that can increase the risk of exposure to high-risk HPV in successive generations of women.

The APC analysis was carried out from 1994 through 2013 for the age group 25–84 (CC) and 20–69 years (CIS) and was based on yearly intervals of age and period. The flexibility of the spline function was determined by the number of knots; the degrees of freedom were considered to be five for each of the spline bases for the three variables (age, period, and cohort) both for CC and for the CIS model. The decision on the number of degrees of freedom was aided through the use of Akaike’s information criterion values. A lower Akaike’s information criterion value suggests a better-fitting model.

All statistical analyses were carried out using the Stata software ver. 14.2 (StataCorp LP, College Station, Texas USA) and Joinpoint software ver. 4.3.1.0 (Surveillance Research Program; National Cancer Institute, Bethesda, Maryland, USA).

## Results

A total of 806 cases of CC and 3022 cases of CIS were newly diagnosed between 1994 and 2013 in Umbria. The 15.9% (128 cases) of carcinomas and the 18% (543 cases) of CIS had arisen in foreign-born women (Table 1). About 80% of these women were from High Migration Pressure countries, especially from Central and Eastern European countries.

**Table 1:**
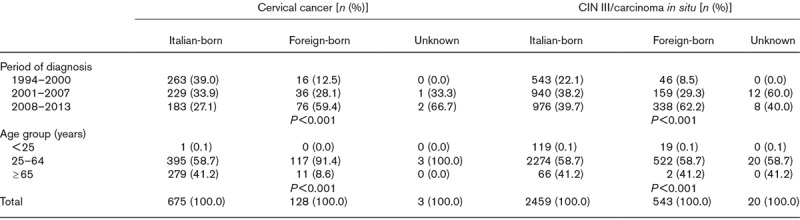
Cervical lesions by period of diagnosis and age group in Italian-born and foreign-born women

Significant differences in the proportion of cases occurring in Italian and foreign women were observed by period of diagnosis and age group both for CC and for CIN III (Table 1). The diagnosis was generally made at a younger age in foreign women than in the Italian-born cohort. The median age at diagnosis of CC was 59 (20–99) and 47 (27–82) years in Italian and foreign women, respectively. For CIN III, the median age at diagnosis was 37 (15–83) years in Italian-born women and 36 (19–68) in the immigrant women.

From 1994 to 2013, a decreasing CC incidence trend was noted in Umbria in women older than 65 years of age [APC = −1.8, 95% CI = −3.9; 0.3], although not statistically significant, whereas in the 25–64-year age group, the incidence remained very stable (APC = 0.1, 95% CI = −1.4; 1.7).

The CIS age-standardized incidence rate increased significantly during the study period: from 12.2 in 1994 to 49.9 in 2013 (APC = 6.4*, 95% CI = 4.8; 8.0 in the entire population; APC = 6.0*, 95% CI = 4.5; 7.6 in the 25–64-year age group).

The age-adjusted incidence rates of cervical lesions by period in the two groups of women are presented in Table 2.

**Table 2:**
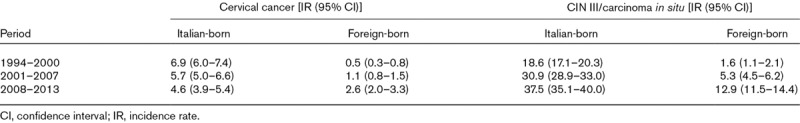
Age-standardized incidence rate per 100 000 of cervical lesions by period of diagnosis in Italian-born and foreign-born women

**Fig. 1:**
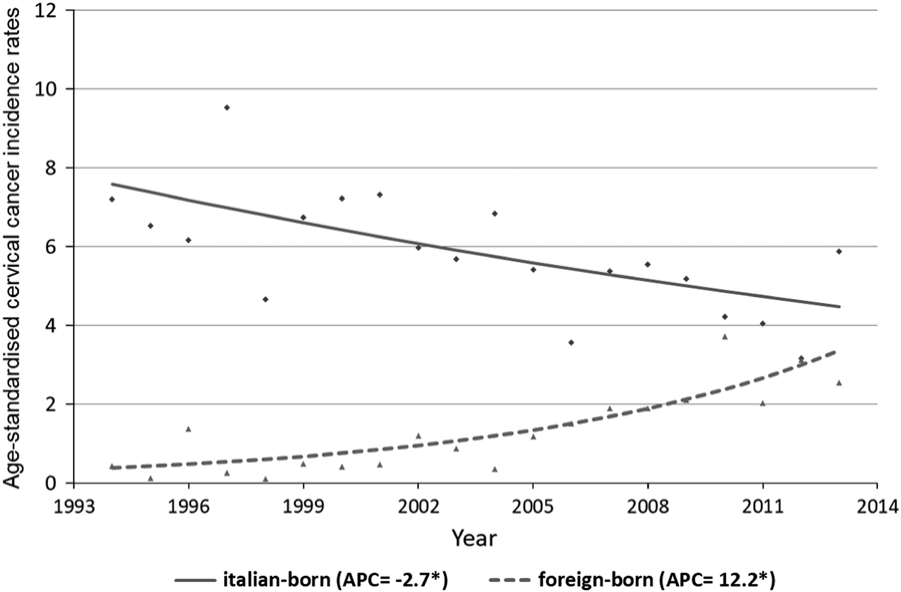
Cervical cancer incidence in Umbria (1994–2013). Observed age-standardized rates and estimated trends by nationality (modeled data results from Joinpoint). APC, annual percent change.

**Fig. 2:**
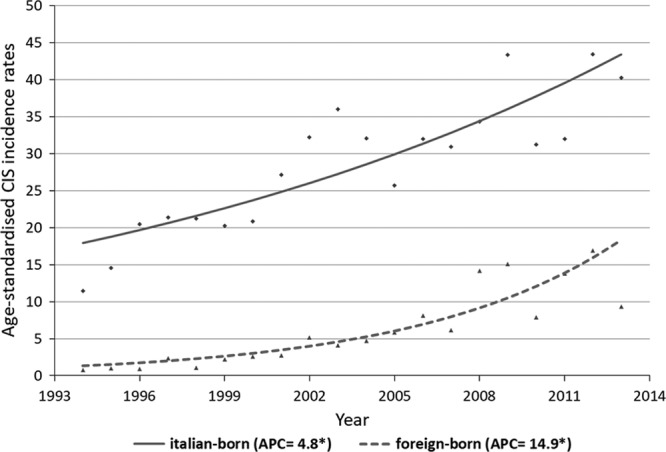
Carcinoma *in-situ* incidence in Umbria (1994–2013). Observed age-standardized rates and estimated trends by nationality (modeled data results from Joinpoint). APC, annual percent change.

For the Italian-born women, the CC incidence rate was 6.9/100 000 in the period 1994–2000 and decreased to 4.6/100 000 in the period 2008–2013. The Joinpoint analysis showed an APC of −2.7* (95% CI: −4.3; −1.1) for Italian-born women between 1994 and 2013. Conversely, the incidence trend of foreign-born women increased from 0.5/100 000 in the period 1994–2000 to 2.6/100 000 in the period 2008–2013, with APC of 12.2* (95% CI: 7.6; 17.0) between 1994 and 2013 (Fig. 1). For CIS, incidence rates showed an increasing trend in both groups, but was much higher in women born abroad. The APCs in the period studied were 4.8* (95% CI: 3.3; 6.3) and 14.9* (95% CI: 10.7; 19.3) in Italian-born and foreign-born women, respectively (Fig. 2).

Between 2001 and 2013, in all women who were 25–64 years old, 46.3% of CC were detected by a Pap smear (organized or spontaneous). The proportion of screening-detected CC in Italian-born women and in foreign-born women did not differ significantly (47.3 vs. 43.8%; *P* = 0.7).

Moreover, in the two groups of women with a preneoplastic lesion, there was a similar (*P* = 0.7) percentage who had participated in the regional prevention program (Table 3).

**Table 3:**
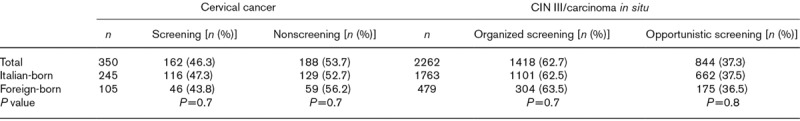
Screening status of women aged 25–64 years with cervical lesions by nationality, Umbria 2001–2013

**Fig. 3:**
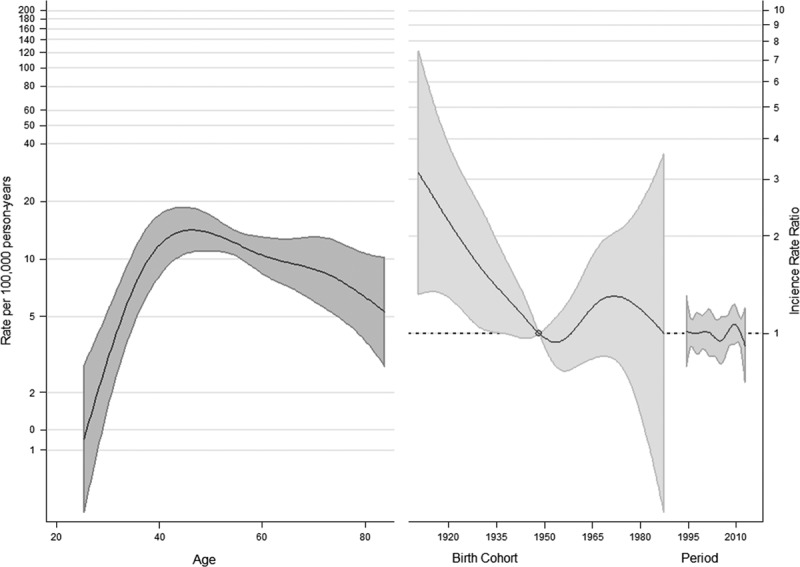
Estimated effects from the weighted APC model for the cervical cancer (*df* = 5). APC, Age–Period–Cohort.

**Fig. 4:**
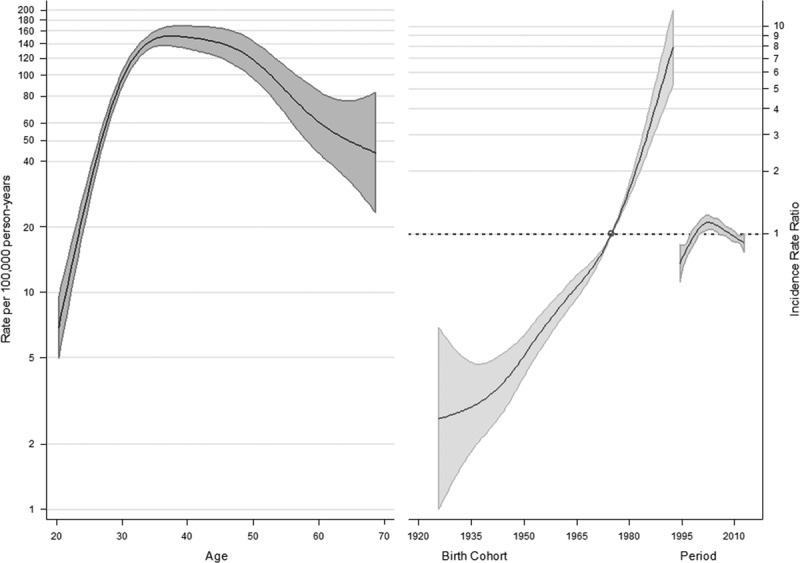
Estimated effects from the weighted Age–Period–Cohort model for carcinoma *in situ* (*df* = 5).

The APC models are shown in Figs 3 and 4. For CC, no considerable changes in period-specific incidence rate ratios (IRRs) were detected until around 2000, when there was a slight decrease, followed by an increase. Nevertheless, in recent years, the trend seems to be declining. An increase in cohort-specific IRRs was observed in women born after the 1950s, preceded by decreasing risks factors in the cohorts born in the first decades of the 20th century. A further decline was observed again in the younger cohorts born after 1970. Age-specific IRR continued to increase from the age of 25 years, flattening after 35–40 years and then slowly declining (Fig. 3).

Considering CIS, a strong increase in cohort-specific risks in subsequent generations was estimated, particularly in women born after 1970. A decline in period-specific IRR was observed after 2000, preceded by a growing trend. Age-specific IRRs flatten early and begin to fall after 35–40 years (Fig. 4).

## Discussion

We examined the trends in the incidence of cervical lesions over a 20-year period in Umbria, where an organized screening program has been in place since 1999.

For CC, a declining trend has been observed in women older than 65 years of age, whereas in the younger group it was stable. Conversely, we found a significant annual increase of 6.4% for CIS.

Our analysis has shown that in Italian-born women, the CC incidence is decreasing, whereas there has been a significant increase in cases diagnosed in immigrant women, especially in recent years. Furthermore, the foreign women, mainly from Central and Eastern European countries, tend to develop CC at a younger age.

As reported by several studies (Tornesello *et al.*, 2014; Di Felice *et al.*, 2015; Visioli *et al.*, 2015), the high CC incidence in foreigners seems to be related both to the high prevalence of HPV infection and the low screening uptake in the immigration country.

At present, CC screening programs are restricted or ineffective in Central and Eastern European states, with several important obstacles, such as low coverage, high number of opportunistic smears, and the absence of follow-up of positive screened women. In addition, only a few countries have actually integrated the HPV vaccination into their national immunization program, mostly performed in local public health centers or school health services. HPV vaccination coverage therefore remains low because of poor acceptance and negative public reactions (Poljak *et al.*, 2013). Indeed, the CC incidence rates in these countries are the highest in Europe and are expected to continue to increase (Vaccarella *et al.*, 2016).

The absence of an organized program in the native country seems to affect the attitude of immigrant women to screening. A variety of sociodemographic and psychological factors can contribute toward the lower participation in screening among immigrants, such as language difficulties, high mobility, difficult working conditions, low perception of risk, and different cultural beliefs (Azerkan *et al.* 2012; Grandahl *et al.*, 2015; Leinonen *et al.*, 2017). Conversely, factors positively affecting participation in screening seem to include young age, good cultural level, a long stay in the host country, being married, and high income (Khadilkar and Chen, 2013; Grandahl *et al.*,2015).

High nonadherence rates were associated with a low socioeconomic status (Rondet *et al.*, 2014, Leinonen *et al.*, 2017) and, in turn, a low socioeconomic status appears to be linked to an increased risk of developing CC (Arnold *et al.*, 2016; Fidler *et al.*, 2016; Ginsburg *et al.*, 2017).

Interestingly, our study showed that the percentage of lesions detected by screening in immigrant women is comparable to that of Italian-born women. In Italy, between 2009 and 2011, a relatively small difference in screening program adherence was found between Italian-born and foreign-born women (46.9 vs. 42.2%) (GISCI. Survey GISCI sulle migranti nei programmi di screening cervicale, 2014). Indeed, the participation of immigrant women seems to be similar to that of Italians during the reproductive age as, in this period of life, women have more contact with health services and healthcare staff, particularly because of pregnancies (Campari *et al.*, 2016).

The increase in the incidence of CC in foreigners can also be explained partly by the high prevalence of HPV infections in their countries of origin. For example, in Romania, a country with high prevalence rates of HPV infection, CC is the first cause of cancer death in women aged between 15 and 44 years, and an infection with a high-risk HPV type is found in about 89% of cases (Pirtea *et al.*, 2016). Di Felice *et al.* (2015) recently reported a higher risk of cervical lesions in women born in high HPV prevalence countries compared with Italian women, confirming that HPV prevalence in the country of origin is a major determinant for the onset of CC.

The age effect shown in our analysis, with upward age-specific IRR until 35–40 years and then slowly declining, was probably influenced by screening activity. The age at diagnosis for CC is related to several factors, such as the age at exposure to HPV, the latency between virus exposure and dysplasia, the immune status, sexual hormones, and the implementation of a screening program. In countries with poor or no screening, the incidence rapidly increases until the premenopausal period, at around 45 years. Conversely, in screened populations, CC incidence rates peak at ~35 years, when the positive effect of removal of precancerous lesions can be observed. However, in both unscreened and screened populations, the CC incidence is approximately constant after the age of 45 years, unless age-specific rates are further distorted by a different effectiveness of screening programs within various periods and cohorts (e.g. a lower uptake in older cohorts) (Vaccarella *et al.*, 2013).

In addition, we observed a progressive decrease in CC risk in the cohorts born in the first decades of the 20th century and the younger cohorts, whereas in women born between 1950 and 1970 a growing risk emerged.

The declining cohort effects in older generations are probably because of improved prevention awareness and increased access to healthcare. However, after the Second World War, many aspects of sexual behavior, including earlier age at first sexual intercourse and multiple lifetime partners, have changed considerably, resulting in a progressive increase in the risk of HPV exposure. Also, several European countries and Japan showed similar increases in CC incidence rates following previous decreases among older generations (Vaccarella *et al.*, 2013).

Interestingly, according to estimated period effects, in our study, a first short beneficial impact of screening seemed to have emerged in the early 2000s, immediately counteracted by the changes in the composition of society, with a progressive increase in the foreign population, which has led to the inversion of the positive trend. However, in recent years, there has been a new decrease in the CC risk, probably because of the increasing adherence to screening by immigrant women.

The favorable effect of screening in preventing the increase in CC risk among the youngest birth cohorts is evident in the Nordic countries, such as Denmark and Finland, where organized screening programs have been in place for a long period and decreases of the formerly high incidence rates were driven by period-specific decreases. Conversely, Central and Eastern European countries did not show any favorable period effect, which likely reflects the lack of adequate screening activities (Vaccarella et al., 2013, 2014).

The sharp increase in preneoplastic lesions emerging from our analysis has been observed in other countries, such as Denmark (Baldur-Felskov *et al.*, 2015; Holst *et al.*, 2016) and the Netherlands (Rozemeijer *et al.*, 2015). This growth in rates is probably related to several factors, including an increased risk of contracting HPV infection because of a higher number of risk factors (e.g. multiple lifetime sexual partners, young age at first sexual intercourse, multi-parity, and oral contraceptive use) and a gradual improvement in diagnostic techniques. Nevertheless, excess use of cervical cytological examinations could also have contributed toward the increase in CIN III rates.

This study has some limitations. First, we used the country of birth for the definition of immigrant status. Precisely, Italian women (according to citizenship) who were born in a foreign country were classified as immigrants, even though the number of such women is presumably rather exiguous.

Second, given the small number of foreign-born women, we did not carry out the APC analysis by categorizing the Umbrian population by nationality. Finally, educational level, socioeconomic factors, or years since immigration are important variables related to participation in screening; hence, efforts to further investigate these factors are on-going for future analyses.

In conclusion, organized screening is the key intervention to prevent CC in our study. Immigration from countries with a high incidence of HPV infections and with poor implementation of preventive policies concealed favorable incidence trends among Italian women. Therefore, it is important to analyze CC trends by ethnicity. We found an increasing incidence of cervical lesions among immigrants from high-risk countries. This trend was associated at least partly with screening participation of immigrant women. The prophylactic vaccination against HPV, offered in Italy since 2008 to all females born since 1996, could contribute considerably toward cancer prevention in the coming years.

## Acknowledgements

This work was supported by the Department of Health, Regional Government of Umbria.

## Conflicts of interest

There are no conflicts of interest.
